# Ultrasound inhibits tumor growth and selectively eliminates malignant brain tumor in vivo

**DOI:** 10.1002/btm2.10660

**Published:** 2024-04-01

**Authors:** Nitsa Buaron, Antonella Mangraviti, Yuan Wang, Ann Liu, Mariangela Pedone, Eric Sankey, Itay Adar, Abraham Nyska, Riki Goldbart, Tamar Traitel, Henry Brem, Betty Tyler, Joseph Kost

**Affiliations:** ^1^ Department of Chemical Engineering Ben‐Gurion University of the Negev Beer‐Sheva Israel; ^2^ Department of Neurosurgery Johns Hopkins University School of Medicine Baltimore Maryland USA; ^3^ Sackler School of Medicine, Tel Aviv University Tel Aviv Israel

**Keywords:** brain tumor, cavitation, glioma, low frequency ultrasound, reactive gliosis

## Abstract

Glioma is one of the most common primary malignant brain tumors. Despite progress in therapeutic approaches, the median survival of patients with glioma remains less than 2 years, generating the need for new therapeutic approaches. Ultrasound (US) is widely used in medical fields and is used as a therapeutic tool mainly for improving the performance of therapeutic entities. In this study, we examined a novel approach using low frequency US (20 kHz) (LFUS) as an independent treatment tool for malignant glioma, since primary studies showed that cancer cells are more susceptible to LFUS than healthy cells. LFUS safety and efficacy were examined in a 9L gliosarcoma‐bearing female Fischer 344 rats. Two LFUS protocols were examined: a one‐time treatment (US1X), and two treatments 24 h apart (US2X). For safety evaluation, rats were monitored for weight change and pain measurements. For efficacy, tumor volume was measured as a function of time and the tumor structural chances were examined histopathologically. LFUS treatment showed rapid inhibition of tumor growth, seen as soon as 12 h after US application. In addition, LFUS was found to affect the tumor structure, which was more extensive (>60% of tumor area) in smaller tumors. In US2X, the tumor tissue was completely destroyed, and an extensive immune response was observed. Importantly, the treatment was highly selective, keeping the healthy tissue surrounding the tumor unharmed. We developed a highly efficient and selective therapeutic protocol for treating malignant glioma with minimal side effects based solely on LFUS.

**Abbreviations**
DAMPs: danger associated molecular patterns
DMEM: Dulbecco's modified eagle medium
GBM: glioblastoma multiforme
H&E: hematoxylin/eosin
LFUS: low frequency US (20 kHz)
MGCs: multinucleated giant cells
RGS: Rat Grimace Scale
US: ultrasound
US1X: US application once
US2X: US application twice with a 24‐h gap

**Translational Impact Statement**

Advancements in the care of patients with malignant gliomas have increased the median survival to approximately 20 months with more long‐term survivors. Clearly, new innovative approaches are needed to significantly improve patient outcomes. We are developing a novel approach utilizing low‐frequency ultrasound (20 kHz) as a standalone treatment or in conjunction with tumor resection to treat intracranial tumors. Results show that this approach is highly effective and selective, eliciting an anti‐tumor immune response while sparing healthy tissue.

## 1 INTRODUCTION

A glioma is a primary brain tumor that originates from glial‐type cells in the brain or spinal cord and are often malignant. The most common and aggressive one is the glioblastoma multiforme (GBM)^1^ making up 59.2% of all gliomas and 50.1% of primary malignant brain tumors.^2^ The prognosis for GBM is often quite poor, and the average survival period with treatment is 14–20 months.^3^ Despite recent progress in therapeutic strategies,^4^, ^5^ the current treatment is still in need of novel therapeutic options.^4^, ^6^ One of the main reasons for the low response rate is the difficulty to achieve complete surgical resection due to the infiltrating nature of GBM cells into the normal brain tissue.^7^, ^8^ In addition, treating the non‐resected margin of the tumor with current adjuvant therapies is not effective since these methods to date are non‐selective and can cause systemic toxicity and damage to normal brain tissue.^9^, ^10^ Local therapeutic strategies that can treat the tumor and its diffusive margin while having minimal toxicity on healthy brain cells, have the potential of significantly improving patient prognosis.

Ultrasound (US), a sound wave with a frequency above the human hearing range (>18 kHz), is used in many different fields (i.e., medical, pharmaceutical and general industries). The classification of US according to high‐ or low‐ frequency US, usually depends on the application itself. Focusing on medical field, for biomedical applications (usually between 20 kHz to 2 MHz), low frequency US (LFUS) devices will be in the range of 20 to 200 kHz.^11^ LFUS has been shown to affect, inter alia, mass transport, enhance release of encapsulated drugs, and facilitate gene therapy.^12^, ^13^, ^14^ In addition, LFUS may be used by itself for selective treatment applications, since it has been reported to induce different biological effects on normal cells than those observed on cancer cells, as can be seen in several studies showing that cancer cells are more susceptible to LFUS.^15^, ^16^, ^17^

Lejbkowicz et al.^15^, ^16^ compared the effect of LFUS on several normal cells (foreskin fibroblast and amniotic fluid epithelial) and cancer cells (breast carcinoma, melanoma, and lung carcinoma) in terms of cell's viability, growth, and functions in vitro. They found lower cell viability, effect on cellular DNA and proteins, and cloning efficiency reduction for the cancer cell lines treated with LFUS. They suggested two predominant bioeffects related to cavitation^18^ as responsible for the observed US effect. First, direct mechanical effects can induce cellular permeability, disrupt cell–cell attachment, leading to cell lysis. Second, sonochemical effects can induce formation of free radicals that can react extracellularly with cellular membranes, and intracellularly induce DNA damage. The selective effect of LFUS was examined in our lab by Azagury et al.,^17^ comparing a human breast epithelial cell line to an ovarian tumor cell line in vitro. They found lower viability for the cancer cell line at all intensities and durations examined. In vivo studies on the KHJJ murine mammary sarcoma tumor model showed that the higher LFUS intensity led to a decrease in tumor growth rate. The mechanisms offered are: acoustic streaming (steady flow of fluid induced by US propagation),^18^ and the bilayer sonophore (absorption of US energy by the lipid bilayer membrane),^19^ attributing the differences in cell membrane structure between metastatic cells (more fluidic) and normal cells (less fluidic) as the cause for the varying susceptibility to US. Although the selective phenomenon is not entirely clear, Bergman E. et al.^20^ findings indicate that cancer cell sensitivity to LFUS (20 kHz) may be related to a common phenomenon occurring in all cancer cells (such as reduced stiffness) regardless of their origin and type, and that stiffness at the level of the individual cell (in oppose to tumor tissues) is the key to selective ultrasound‐induced cell death.

The results of these studies suggest that biomechanical‐based selective treatment on malignant glioma could be achieved using LFUS alone. We focused on the conditions that were found experimentally safe for healthy brain tissue. The effect of LFUS was examined with no additional substances (e.g., microbubbles, sonosensitizers, or drugs). This study presents proof of concept of LFUS as monotherapy and the great potential of LFUS‐based therapy.

## 2 RESULTS

### 2.1 Reduction in glioma cells viability in vitro by LFUS

**FIGURE 1:**
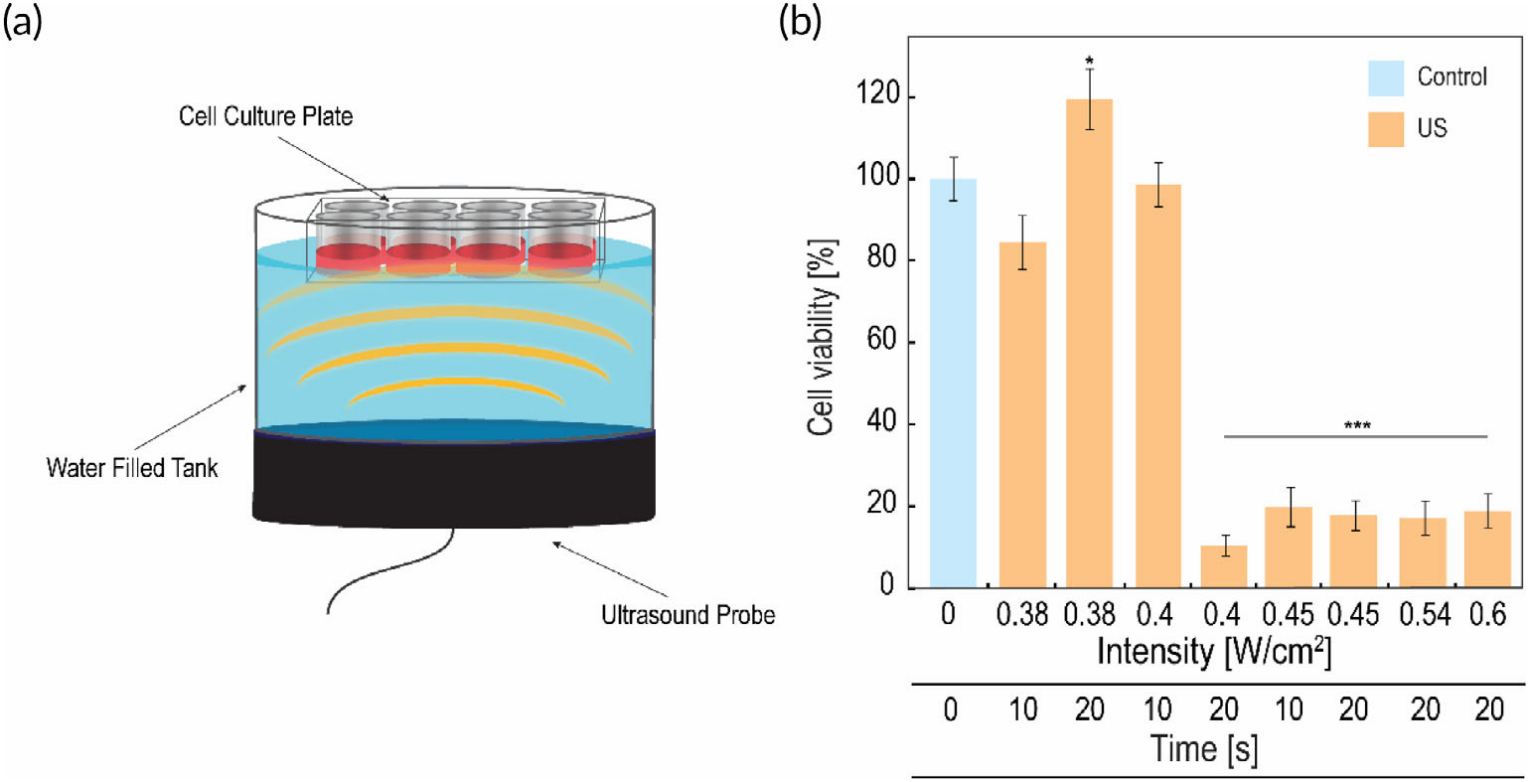
LFUS reduced cell viability in C6 cells: (a) Schematic drawing of the in vitro LFUS experimental set‐up, the cell plate was placed on top of a water‐filled tank above the ultrasonic microplate probe. (b) C6 cell viability after exposure to different LFUS conditions compared to control (no US exposure). MTT assay was performed 1 h after insonation. Bars represent mean ± SEM based on 3 individual experiments; *p < 0.05, **p < 0.01, ***p < 0.001 in comparison to the control by *t*‐test of two samples assuming unequal variance.

Sensitivity of C6 glioma cells to 20 kHz LFUS was examined in vitro in order to assess the therapeutic potential of this frequency (Figure 1b). LFUS intensities up to 0.4 W/cm^2^ for 10 s exhibited high cell viability of more than 80%. However, an intensity of 0.4 W/cm^2^ for 20 s and greater intensities exhibited very low cell viability of under 20% (*p* value <0.001). The sensitivity of healthy brain cells was not examined in in vitro models due to several reasons. First, the available neuronal cell lines are tumor‐derived and often require the addition of environmental signals to display better neuronal specifications.^21^ Our hypothesis is based on the difference in mechanical properties of cancer versus non‐cancer cells. Thus, tumor‐derived cell lines as a model for healthy brain cells are problematic. Second, primary neuronal cell cultures are difficult to prepare and isolate, and the number of cells is limited since they do not divide,^21^, ^22^ making this model costly and impractical for this study. Therefore, we pursue the examination of our hypothesis with a rodent in vivo model.

### 2.2 Safety of LFUS conditions in vivo

**FIGURE 2:**
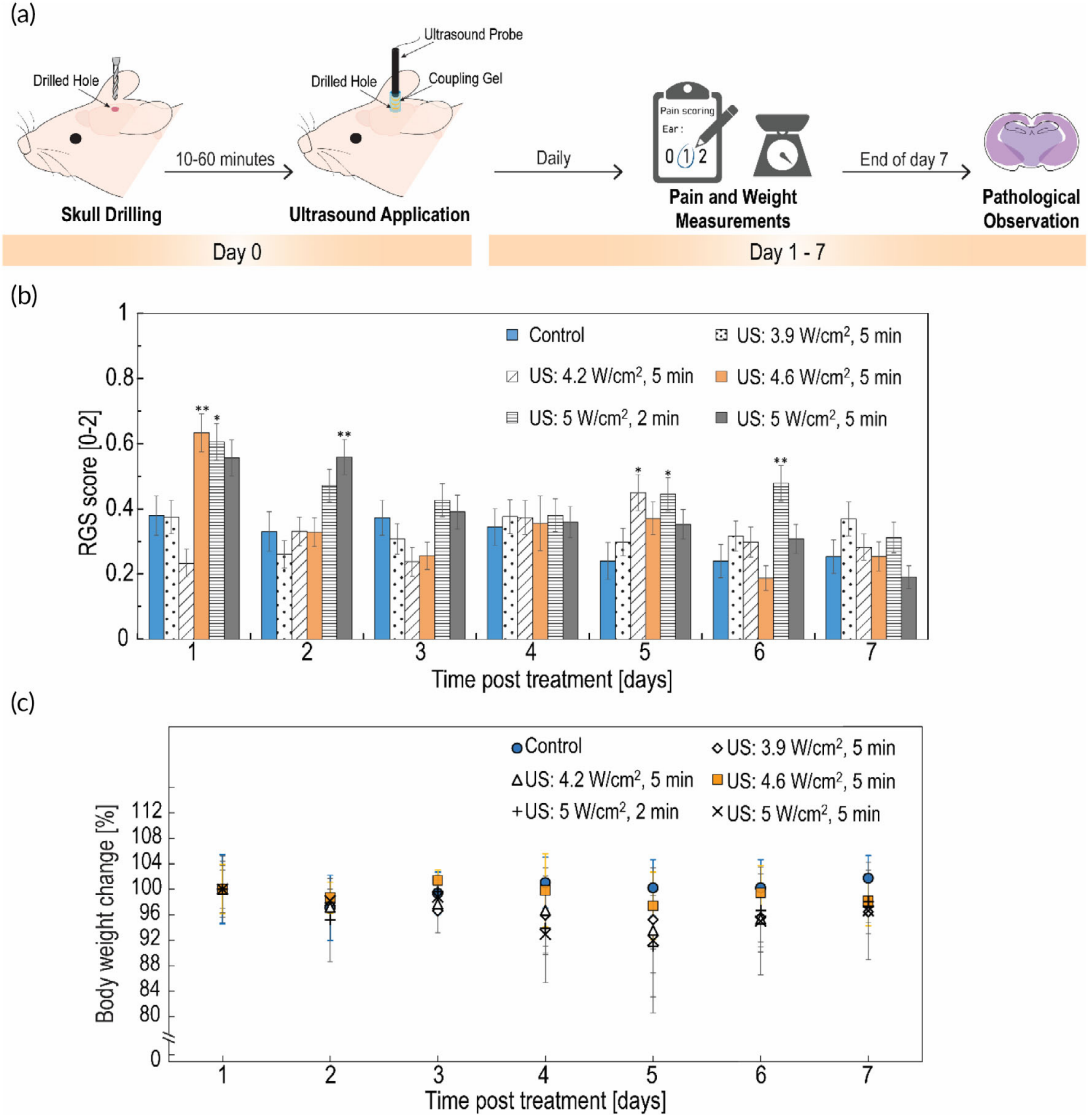
Safety assessment of the in vivo LFUS conditions: (a) A schematic workflow of the safety experiment: the skull was drilled and LFUS at intensities of 3.9, 4.2, 4.6, 5 W/cm^2^ for 5 min and 5 W/cm^2^ for 2 min was applied. Pain scores and weights were measured daily for 7 days, and on day 7 the brains were examined by pathological observation. (b) Quantification of spontaneous pain at different time points post‐insonation; Bars represent RGS score mean ± SEM RGS; *p < 0.05, **p < 0.01 compared to control by Dunnett's case‐comparison post‐hoc test (one‐way). (c) Weight changes at different time points post‐insonation. Bars represent mean ± SD.

20 kHz LFUS application was further examined in vivo. An in vivo LFUS apparatus was assembled and US conditions for healthy brain tissue were evaluated in terms of animals' pain measurements, weight change, and histopathological assessment (Figure 2a). By examining average spontaneous pain through facial expression scoring, we found that pain values were relatively low (Figure 2b). All values obtained were between 0 and 1, which means moderate pain or less. In the control group the score values were around 0.3 at all the time points. The same pattern can be seen for 3.9 W/cm^2^. In the group of 4.2 W/cm^2^, all the score values were as the control except for day 5 which the value was higher and statistically different. In the group of 4.6 W/cm^2^, the score in the first day was ~0.6, and in the next days the values were as the control. In the group of 5 W/cm^2^ for 2 min, score values higher than the control were observed on days 1, 2, 5, and 6. In the group of 5 W/cm^2^ for 5 min, score values higher than the control were observed on days 1, and 2. Overall, the highest values were observed in the first 2 days, and on day 5, meaning that the main pain effect was in these days. The highest pain effect (more days with values higher than the control) were found for the groups of 5 W/cm^2^, even though all values were found to be on the scale of moderate pain and less. In addition, as can be seen in Figure 2c, animals maintained relatively stable weights throughout the study. In the control group the body weight was slightly decreased on day 2 (~3%) and then gradually increased reaching 2% of increase on day 7. In the groups of 3.9 W/cm^2^ and 4.6 W/cm^2^ weight was relatively stable, and in the group 4.2 W/cm^2^ the same pattern was observed except for day 5 were 6% of decrease is observed. For the groups of 5 W/cm^2^ a decrease in weight of 8%–9% was observed on days 4 and 5, this correlates with higher pain values observed on days 5 and 6. Most of the changes were observed on days 4 and 5 and are in the range of ~10% from the initial weight. Histopathological evaluation of control rat's brains found common features at the drilling site, presenting superficial injury only in gray matter. Histological evaluation of US intensities up to 4.6 W/cm^2^ for 5 min found similar features as the control group. At intensity of 5 W/cm^2^ for 2 and 5 min, a deeper damage was observed (also in white matter), indicating on additional damage in the brain tissue compared to control (pathological images not shown). To conclude, we found that US irradiation of 5 W/cm^2^ for 2 and 5 min were found to have toxicity mainly observed by histopathology features, and US irradiation of 4.6 W/cm^2^ and down were found to be safe at all methods examined. In order to maximize the LFUS therapeutic effect, we continued the investigation with the intensity of 4.6 W/cm^2^, which is the upper limit of the safe range for healthy brain tissue.

### 2.3 LFUS inhibits tumor growth and leads to tumor elimination in vivo

**FIGURE 3:**
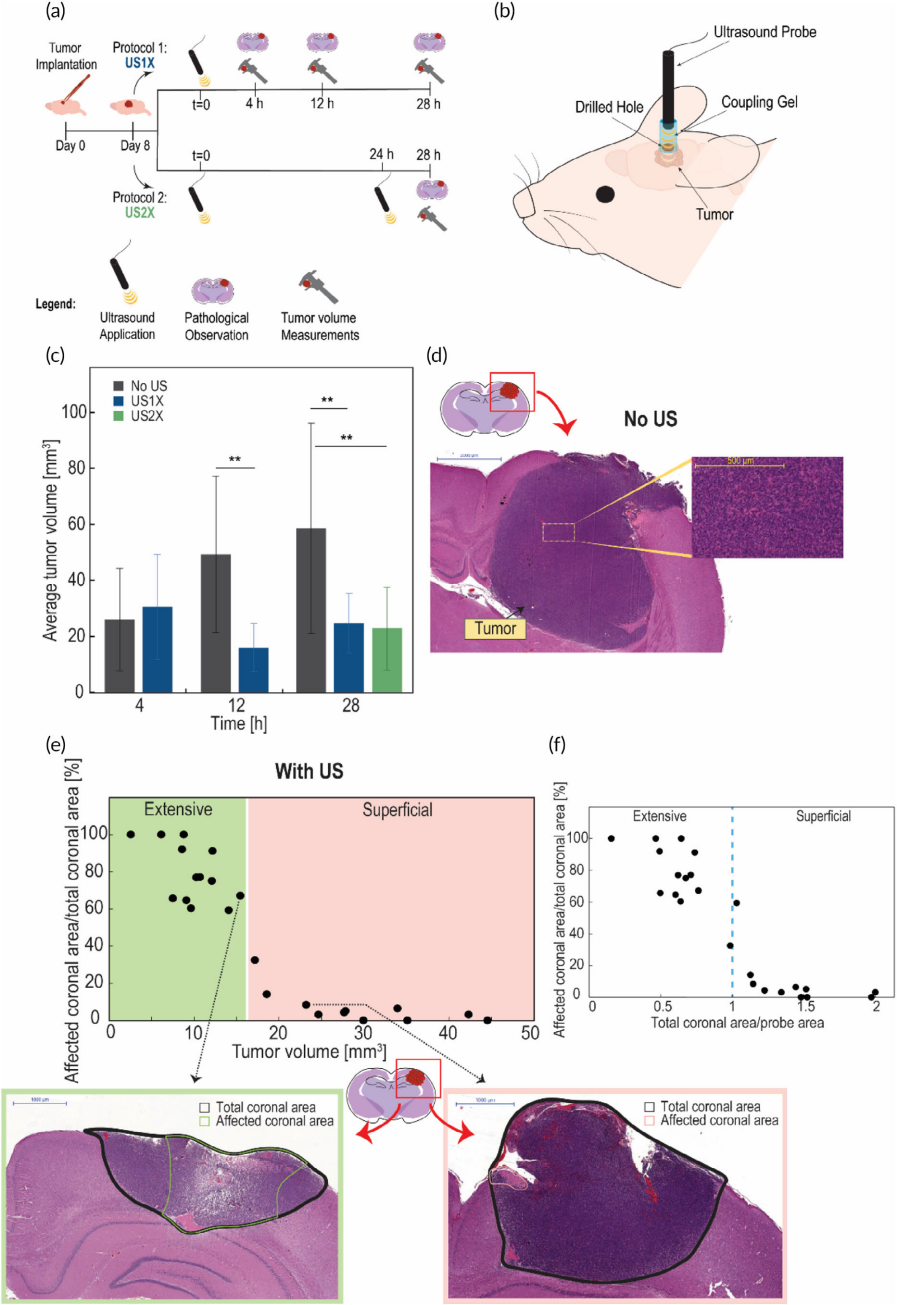
Inhibition of tumor growth by LFUS application. (a) A schematic workflow of the experiment: the LFUS effect on intracranial 9L gliosarcoma tumor in vivo. Two LFUS protocols were examined: one‐time treatment (US1X) and two treatments 24 h apart (US2X) as illustrated; and one control protocol (untreated group). (b) Illustration of the in vivo experimental setup of LFUS (20 kHz) application: the LFUS probe is immersed in a gel‐filled cylinder, 2 mm above the drilled hole in the skull of a tumor bearing rat. (c) Effect of LFUS (4.6 W/cm^2^ for 5 min) on 9L gliosarcoma tumor growth: Average tumor volume as a function of time post‐LFUS treatment for the different experimental groups (8 ≤ *n* ≤ 14), control group (no US), group treated with US1X, and group with US2X; *p < 0.05, **p < 0.01, *** < 0.001 in comparison to the control group (*t* = 0) by t‐test of two samples assuming unequal variance. (d) Coronal section of intracranially implanted 9L gliosarcoma tumor in a F344 rat, showing the tumor region (left image) and enlargement of the marked area in the tumor (right), 9 days after tumor implantation (control group). Scale bar = 2000 μm (left image) and 500 μm (right image). (e) The dependence of LFUS application on tumor volume: The percentage of the affected coronal area by LFUS irradiation from the total mid‐coronal section tumor area as a function of the tumor volume for all LFUS experimental groups, which were histologically evaluated (4 groups: 4, 12, 28 h US1X, and 28 h US2X; *n* = 6 per group). Coronal sections of representative rat brains which were analyzed, one from the extensive effect region (marked in green) and one from the superficial effect region (marked in pink). Scale bar = 1000 μm. (f) The dependence of LFUS application effect on the tumor and LFUS probe dimensions: The percentage of the affected coronal area by LFUS irradiation from the total mid‐coronal section tumoral area as a function of the ratio of the total coronal tumor area to the LFUS probe surface area for all LFUS experimental groups which were histologically evaluated (4 groups: 4, 12, 28 h US1X, and 28 h US2X; *n* = 6 per group). Blue dashed line represents the transition between extensive and superficial regions.

We examine the effect of 4.6 W/cm^2^ LFUS irradiation on an intracranial 9L gliosarcoma tumor's volume growth and structural changes in vivo. Two LFUS protocols were used, a one‐time treatment (US1X) and two treatments, 24 h apart (US2X) (Figure 3a,b). For efficacy evaluation, tumor growth was examined by measuring the tumor volume at different time points after LFUS application and then comparing it to the untreated group (Figure 3c). For the untreated group (no LFUS), rapid tumor growth was observed. Average tumor volume doubled between the time points of 4 and 12 h and increased by an average of ~1.2 fold between 12 and 28 h. It is important to note that the experiment was done 8 days after tumor inoculation (which is at a very progressed stage of the tumor growth^23^, ^24^) and therefore rapid increase in tumor growth in the control group is expected. The untreated group and the US1X group had similar tumor volumes at 4 h. However, by 12 h, the volume of the US1X group had decreased 3‐fold (*p* value <0.01) compared to the untreated group, and 2‐fold compared to the average volume of the two groups at the 4‐h time‐point. A similar observation was found at 28 h, with a 1.2‐fold significant decrease (*p* value <0.01) in the US1X group compared to the untreated group. The average tumor volume of the US2X group)at 28‐h(was similar to the US1X group and showed a 1.2‐fold significant decrease (*p* value <0.01) compared to the untreated group.

Tumor tissues were examined histopathologically in order to assess the structural changes resulting from the two LFUS protocols compare to control. First, the characteristics of the control group tumor tissue were evaluated (Figure 3d). The tumors consisted of a compact cellular mass, composed of uniform dense population of cancerous cells without any necrosis. The margins of the tumor tissue were clear and sharp, with no evidence of edema or penumbra in the adjacent tissue. When examining the tumor tissue of the LFUS treated groups, a very clear difference in terms of structural change was observed in tumors with different volumes (Figure 3e). We found that the LFUS effect on the tumor (i.e., the observed structural changes) could be roughly divided into two distinct categories. The first category included tumor volumes higher than 16 mm^3^, in which the LFUS effect was superficial with less than 40% of the tumor affected. The second category included tumor volumes smaller than 16 mm^3^, where the LFUS effect was extensive, covering between 60% and 100% of the tumor area.

It is important to note that even though the structural changes were minor for volumes greater than 16 mm^3^, an inhibition of tumor growth was observed. In addition, the defined transition volume of 16 mm^3^ is a value which is dependent on, and relevant to, the LFUS condition used in this study. In order to achieve a more general description of the results that can be applied to a broader range of tumor volumes and LFUS conditions, we created a dimensionless graph (Figure 3f). The area that best represents the tumor volume is the coronal section since it consists of both length and depth of the tumor. The coronal tumor area was divided by the LFUS probe surface area, and the coronal affected area percentage was plotted as a function of this ratio (Figure 3f). The distribution obtained (as also shown in the volume dependent graph (Figure 3e) can be divided into two regions, one of extensive effect and another of superficial effect. The transition between the regions is a ratio of 1, observed when the tumor coronal area is equal to the probe surface area. When the coronal area is smaller than the LFUS probe area (ratio smaller than 1), the effect of LFUS is extensive, and when it is larger (ratio larger than 1) the effect is superficial.

**FIGURE 4:**
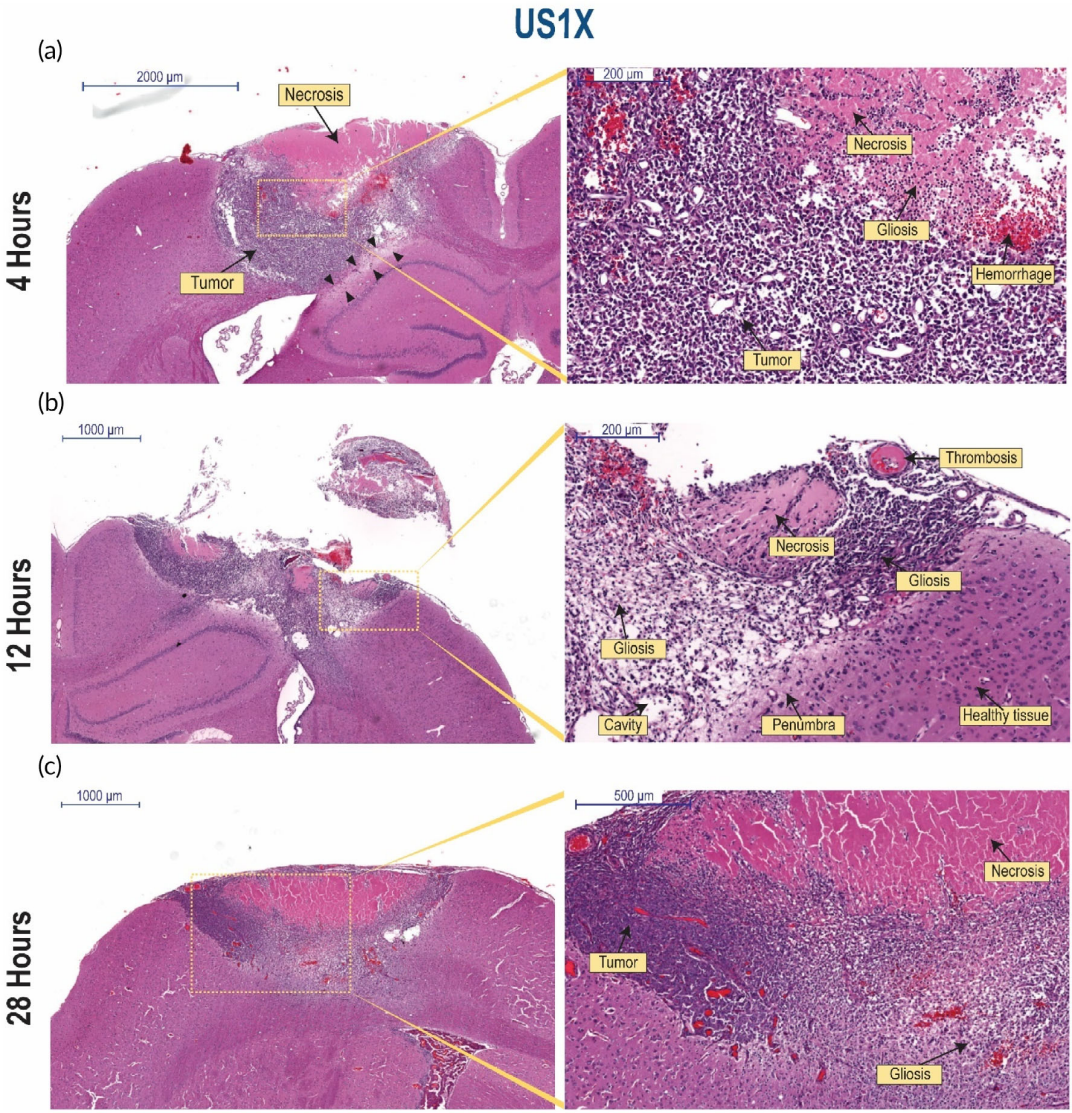
Histopathological evaluation of the resultant structural changes from treating the tumors with US1X at different time points post application. (a) Different magnifications of a coronal brain section from a rat bearing a 14 mm^3^ intracranial 9L tumor 4 h post‐insonation. Histological characteristics are marked within the images. Scale bar = 2000 μm (left image) and 500 μm (right image). (b) Different magnifications of a coronal brain section of rat bearing a 3 mm^3^ 9L tumor 12 h post‐insonation. Histological characteristics are marked within the images. Scale bar = 1000 μm (left image) and 500 μm (right image). (c) Different magnifications of coronal brain section of rat bearing a 9 mm^3^ 9L tumor 28 h post‐insonation. Histological characteristics are marked within the images. Scale bar = 1000 μm (left image) and 500 μm (right image).

**FIGURE 5:**
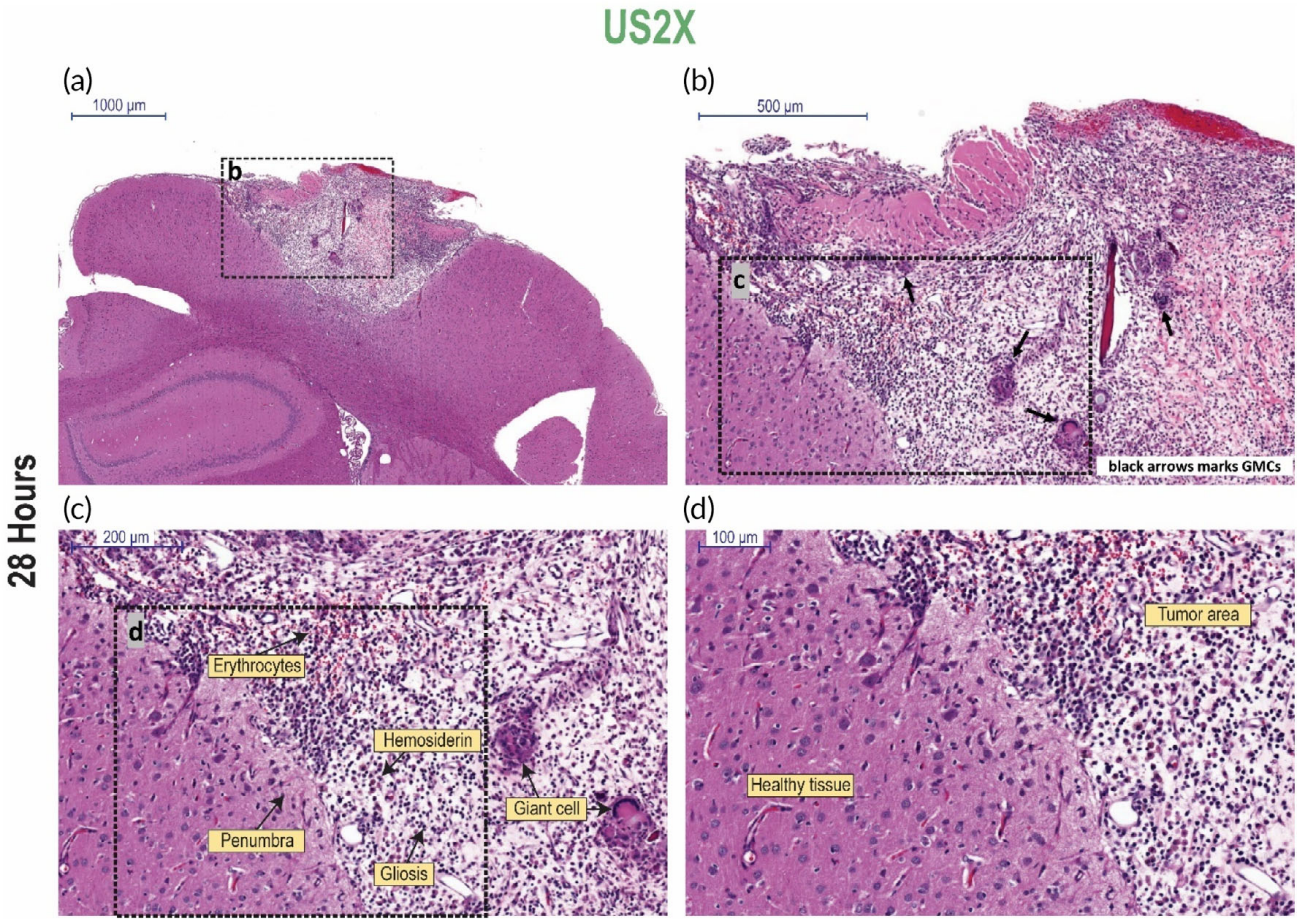
Histopathological evaluation of the resultant structural changes from treating the tumors with US2X, 28 h after the first application. (a–d) Different magnifications of a coronal brain section of rat bearing a 6 mm^3^ 9L tumor after treatment with US2X (magnifications as marked in the images). Histological characteristics are marked within the images. Scale bar = 1000 μm (image a), 500 μm (image b), 200 μm (image c), 100 μm (image d).

A comprehensive description of the structural changes observed in the tumor volumes that were smaller than 16 mm^3^ are presented in Figure 4 and Figure 5. For the US1X group, we examined the tumor tissue 4, 12, and 28 h after LFUS treatment. At 4 h, a large necrotic area was found close to the brain's surface (Figure 4a). The necrotic features included an area of no cells, ghost cells (cells without nuclei), necrotic cells, and erythrocytes. In addition, inflammatory cells were noted. Since the location of the necrosis was at the surface of the tumor, this is likely caused by external exposure from the LFUS radiation rather than the malignancy of the tumor.^25^ In addition, at the margin of the tumor area, under the necrosis (outlined with black arrowheads), the penumbra region can be seen with spongiosis (vacuolation). At 12 h (Figure 4b), most of the tumor area was replaced with necrosis and glial reaction. An extensive gliosis was found along with thrombosis, edema, hemorrhage, and mineralization (Supplementary Figure S1). In addition, cavities were formed as a result of clearance of the necrotic tissue by the glial reaction. The tumor was surrounded by the penumbra region with adjacent healthy neurons. These exciting results indicate that 12 h post US1X, the entire tumor was destroyed, while the surrounding healthy tissue was left unharmed. In tumors in which the volume was close to 16 mm^3^ (Supplementary Figure S1), small areas of intact tumor tissue still remained on the margins located at the sides of the tumor. Investigating the tumor tissues 28 h after LFUS treatment (Figure 4c), we observed the same pattern, in which most of the tumor area was replaced with necrosis and glial reaction. In the center of the tumor, a necrotic area involving over 90% of the tumor was found. At the margins of this area, infiltration of glial cells was noted, clearing the necrotic cells.

Remarkable results were seen in the group that was treated with US2X (Figure 5). Results show that the entire tumor area was replaced with necrosis and extensive glial reaction (Figure 5a). In some samples, mineralization was also found (Supplementary Figure S1). The necrotic reaction was very small due to the infiltration of glial cells. No remnants of tumor cells were noted. Extensive gliosis was identified along with multinucleated giant cells (MGCs) engulfing the necrotic tumoral tissue (Figure 5b,c). The extensive glial reaction and the presence of MGCs is indicative of the high rate of elimination of the necrotic tissue. In addition, erythrocytes and hemosiderin were noted in the necrotic area, indicative of hemorrhage. Moreover, cavities were formed, probably due to necrotic tissue clearance by the glial reactive cells (Figure 5c). Surrounding the tumor tissue, a penumbra region was found followed by healthy neural tissue (Figure 5c,d). These results indicate that the entire tumor was eliminated by the LFUS treatment, while the healthy tissue surrounding the tumor remained unaffected.

## 3 DISCUSSION

We investigate the potential of LFUS (20 kHz) to reach maximal tumor destructive effect while having minimal toxicity on the surrounding healthy tissue. We chose a low frequency, high intensity, and a relatively long duration while maintaining safe parameters for healthy tissue, and took into account the experimental conditions (e.g., the probe diameter needed for covering the maximal tumor area for direct LFUS exposure).

The sensitivity of glioma cells to LFUS was examined in vitro and in vivo. The examination of two LFUS protocols in vivo (Figure 3a–c) indicates that these protocols, which proved to be safe for healthy brain tissue (Figure 2), significantly inhibited tumor growth in a very short time period. Additionally, the two LFUS protocols showed the same inhibition rate (Figure 3c). It is possible however, that the 4‐h time‐point after the second application for the US2X group was too short to observe additional decrease in tumor volume, as seen in the US1X group which started showing significant decrease 12 h post LFUS application. A follow‐up study that will include long‐term (1–2 weeks) observation of tumor volume post‐LFUS treatments can reveal an additional decrease in tumor volume and shed light on the differences between the two protocols in terms of tumor volume reduction. Tumor inhibition is also correlated with our in vitro study (Figure 1), in which increasing LFUS intensities and durations led to a decrease in C6 cell viability. This correlates with previous studies showing that treatment with US can decrease cancer cell viability and affect intracellular processes leading to toxic effects in vitro.^26^, ^27^ Our present data shows that glioma cells are highly sensitive to LFUS, suggesting that this frequency has great therapeutic potential.

Assessment of the structural changes of the tumor tissue (Figure 3d, Figure 4 and Figure 5) indicates that morphological reaction development was very fast,^28^ transforming from acute to subacute within 4–28 h. This fast reaction development was correlated with the rapid inhibition of tumor growth seen 12 h after LFUS application. Tumor growth inhibition may be due to multiple mechanisms which can be described through the observed tumoral tissue structural changes. First, hemorrhage, hemorrhage‐related signs (e.g., erythrocytes, hemosiderin), and edema are indicative of damage to the tumor blood vessels,^29^ temporal or permanent, which in our study was probably caused by LFUS. This could lead to a reduction in blood flow to the tumor area, inhibiting the vascular supply and thus causing growth inhibition. It was previously reported that US (1 MHz and microbubbles/contrast agent) can reduce blood flow in tumor.^30^, ^31^ These leaky, fragile tumor blood vessels may be more sensitive to LFUS than healthy tissue blood vessels. Second, tumor cell damage characterized by necrosis and associated mineralization may occur due to permanent cell membrane disruption caused by LFUS, leading to cell lysis and consequent necrosis. This can be due to the formation of irreversible pores in the cell membrane caused by sonoporation. Sonoporation and the resultant tumor cell damage can occur as a result of several US effects, both sonochemical and sonomechanical related. Sonochemical effects involving the collapse of a cavitation bubble can lead to the formation of free radicals due to thermal dissociation of water. These radicals can react with the cell membrane and lead to its permeability.^15^, ^16^ Sonomechanical effects can be cavitation related and non‐cavitation related. Studies suggest that mainly transient cavitation^32^, ^33^, ^34^ effects, involving the collapse of a bubble (e.g., shear stress, shock waves, and microjets), may lead to structural deformation of the cell membranes, consequently leading to its permeability which can be permanent and lead to cell death.^35^, ^36^ Acoustic streaming may also play an important role in cavitation side effects on membrane permeability.^18^ Additional sonomechanical effect may be the “bilayer sonophore” model,^19^ which suggests that US energy can be absorbed by the lipid bilayer membrane, either in cavitational or sub‐cavitational^37^ US intensities. This induces mechanical stresses and strains in the space between the membrane lipid layers which enhances the membrane permeability.^19^

The induction of a glial reaction was observed only in the groups that were treated with LFUS and not in the untreated groups (Figure 3d, Figure 4 and Figure 5). Extensive gliosis was observed when LFUS was applied twice (Figure 5). These findings indicate that LFUS can probably induce an anti‐tumor immune response. Necrosis and blood vessel disruption can trigger the immune system, consequently stimulating an inflammatory response.^38^ Microglia and macrophages, the main immune cells involved in the glial reaction, rely on microenvironmental cues to trigger their polarization state, and their M1 or M2 phenotype, associated with pro‐inflammatory or anti‐inflammatory functions, respectively.^39^ The solid tumor microenvironment is immunosuppressive, and promotes M2 polarization, which promotes disease progression in dysfunctional tissue.^40^ When US induces necrosis, the necrotic cells release molecules such as cytokines and danger‐associated molecular patterns (DAMPs)^38^ into the tumor microenvironment. These cues trigger microglia/macrophage into a M1‐phenotype pro‐inflammatory polarization state.^41^, ^42^ Upon US stimulation, microglia and macrophage present in the solid tumor may change their polarization into the M1‐phenotype and be recruited for tumor elimination, as it is now recognized as a damaged area. US stimulation causes an anti‐tumor response of the immune system in the form of a glial reaction, which also involves infiltration of glia cells from the healthy neural tissue into the tumor area. This may explain why application of US2X was more effective than US1X; the tumor's microenvironment is enriched with more pro‐inflammatory cues which stimulate an additional anti‐tumoral immune response, consequently leading to the efficient elimination of the tumor from the brain. Interestingly, the immune response after LFUS application in the condition we used was very rapid and extensive. One of the main advantages of this type of therapy, which involves the recruitment of the immune response, is the prevention of side effects related to necrosis (e.g., an increase in intracranial pressure^43^) as the affected area is completely eliminated from the brain parenchyma, indicating a potentially promising anti‐tumor treatment.

A broad range of tumor volumes were found at each time point for the untreated group (Figure 3c), indicating the vast variation in tumor growth between animals. This point was a limitation of this study since the same conditions of LFUS were applied on different initial volumes. It is important for future examinations to adjust different LFUS protocols according to initial tumor volume. Indeed, analyzing the treatment effectiveness in terms of the LFUS probe and tumor dimensions (Figure 3d–f) can be used as a strategy for adjustment of LFUS protocols. LFUS effect is dependent on both tumor depth and length, thus LFUS conditions needs to be chosen accordingly for optimization. The effect of LFUS on the length, or the transverse tumor area, is greatly dependent upon the probe surface area. The more that the tumor area is exposed, the greater the probability for LFUS‐tissue interaction. In our experiments, both the probe diameter and skull burr hole were 3 mm, allowing the probe to cover the maximal area possible for direct exposure to LFUS. The effect of LFUS on the depth is greatly dependent upon the frequency. The lower the frequency, the deeper the probe penetration depth. Since we used a frequency which is at the lower range of the US scale, deep penetration of the tumor was expected. Indeed, as shown in the results, maximizing US‐tissue interactions by using LFUS conditions to affect most of the tumor volume (i.e., ratio <1) can lead to an extensive therapeutic effect. Evaluating the coronal dimensions using diagnostic tools (such as MRI) and choosing the appropriate LFUS probe accordingly can be a potential therapeutic approach for reaching maximal LFUS effect. Additional options include using multiple small probes and applying LFUS at different locations of the tumor to ensure optimal coverage. As a future non‐invasive therapeutic approach, focused US may be a possible option.

## 4 CONCLUSIONS

In summary, our study shows high selectivity accompanied with high efficacy when LFUS monotherapy was used as a primary treatment tool for treating malignant glioma in vivo. LFUS applied in conditions which were shown to be safe to healthy tissue, induced rapid inhibition of tumor growth. In addition, we demonstrated that over time, insonation specifically damaged the tumoral tissue, resulting in necrosis confined by a gliotic reaction. When LFUS was applied twice, the glial reaction was more progressed and extensive, with all of the tumor tissue destroyed and cleared. One of the most important observations was the minimal neurotoxicity and high selectivity of the LFUS treatment, while the tumor tissue was destroyed by the LFUS treatment the healthy tissue surrounding the tumor tissue was left unharmed. These results may pave the way for a singular therapeutic approach for treating malignant glioma by adjusting LFUS protocols according to tumor volume. Additional therapeutic strategies may include using LFUS as an adjuvant therapy after surgical resection. Intracavity LFUS irradiation can be applied in order to eliminate in a selective and effective manner the non‐resected margin of the tumor and diffusive tumor cells from the healthy brain tissue. An additional benefit of using LFUS as a therapeutic tool is that it can penetrate deep into the tissue, and therefore can be used for treating deep seated malignant tumors or intracranial metastasis. To our knowledge, this is the first study performed on glioma in vivo using LFUS with no additional therapeutics.

## 5 MATERIALS AND METHODS

### 5.1 The effect of low frequency US on cell viability in vitro

C6 rat glioma cells were seeded in a 24‐well plate (described in “Supplementary Materials”). The plate was placed on top of a water‐filled reservoir, 3 cm above the ultrasonic 13 cm microplate horn (20 kHz Misonix—ultrasonic liquid processor S4000‐010, max intensity 600 W) (Figure 1a). US was applied at intensities of 0.38–0.6 W/cm^2^ in a continuous mode for durations of 10 or 20 s. After US application, cells were incubated for 1 h and MTT (Thiazolyl Blue Tetrazolium Blue) viability assay was performed (described in “Supplementary Materials”). Results were expressed as viability percentage relative to untreated cells.

### 5.2 Animals

Female Fischer 344 rats were used (described in “Supplementary Materials”). Female Fischer 344 rats were chosen for this set of studies since the 9L tumor is syngeneic in this strain. We have not found a difference in tumor growth rate between males and females using this tumor line. The 9L tumor model has been used as the basis for the preclinical work for both Gliadel, FDA‐approved chemotherapeutic eluting wafers implanted intracranially for the treatment of malignant glioma, and OncoGel, a chemotherapeutic eluting thermosensitive paste.^44^, ^45^, ^46^ While no tumor model fully recapitulates human malignant glioma, the 9L model has an aggressive growth pattern with short median survival and is notoriously difficult to treat. The policies and guidelines of the Johns Hopkins University Animal Care and Use Committee were followed throughout the study under an Animal Care and Use Committee‐approved protocols (animal protocol numbers: RA19M38 and RA20M97), all animal studies meet ARRIVE guidelines and include randomization as well as sample size justification and statistical power analyses.

### 5.3 Intracranial burr hole placement

Rats were anesthetized (described in “Supplementary Materials”). The head was shaved and prepared with alcohol and prepodyne solution (DeLaval Inc.), and a midline scalp incision was made, exposing the sagittal and coronal sutures. A 3 mm burr hole was made in the skull, 5 mm posterior and 3 mm to the right of the bregma, using an electric drill.

### 5.4 Low frequency US in vivo experimental setup

Rats were placed in a custom‐made stand where their head was stabilized, and the stand could be lifted up and down in order to fix the height of the US probe. A cylinder (diameter of 1.5 cm) was placed on top of the head and the US probe was inserted into the cylinder (filled with US gel, described in “Supplementary Materials”) over the burr hole. The probe height was fixed at 2 mm above the drilled hole in the skull (Figure 3b). 20 kHz US (Q125 Sonicator, Qsonica L.L.C, Newtown, CT, USA) with a probe tip diameter of 3.2 mm was used. The US was applied in continuous mode.

### 5.5 Safety and biocompatibility of low frequency US application

Rats (6 per group) were placed in a custom‐made stand (described in “Low frequency US *in vivo* experimental setup”). US was applied in continuous mode 10–60 min after the skull was drilled and under full anesthesia. The applied US conditions were: 5 W/cm^2^ for 5 min, 5 W/cm^2^ for 2 min, 4.6 W/cm^2^ for 5 min, 4.2 W/cm^2^ for 5 min, 3.9 W/cm^2^ for 5 min. The skin was then closed with tissue adhesive (3 M Vetbond™). As a control, the same surgical protocol was followed, apart from the US application. All surgical procedures were performed using standard sterile techniques. Rats were monitored 7 days for weight change and pain measurement. Animals were then euthanized, and brains were placed in 4% paraformaldehyde for at least 24 h and sectioned for further examination.

### 5.6 Quantification of pain using the Rat Grimace Scale (RGS)

Rats were monitored for quantification of pain using the RGS method.^47^ Accordingly, rats were monitored daily for 7 days, for pain‐related facial “action units” as defined by the RGS (described in “Supplementary Materials”). The variability within each group was described by standard error of the mean (SEM).

### 5.7 Histopathological evaluation of safety and biocompatibility of low frequency US application

After pain evaluation, the brains were removed carefully, fixed using 4% paraformaldehyde, and embedded in paraffin to obtain coronal sections of 10 μm. Sections were than stained with hematoxylin/eosin (H&E) for histological observation to evaluate tissue damage. Brain tissue was examined in a double‐blinded manner.

### 5.8 The effect of low frequency US on 9L gliosarcoma tumor mass

Eight days after 9L gliosarcoma tumor implantation (surgery is described in “Supplementary Materials”) under anesthesia, the incision was opened and the burr hole was located. Tumor bearing rats were divided into groups (8–14 rats per group) and were placed in a custom‐made stand (described in “Low frequency US *in vivo* experimental setup”). The applied US conditions were 4.6 W/cm^2^ for 5 min. The groups included: (1) Control group‐ no US treatment; (2) One‐time treatment group‐ protocol of one US application (US1X); (3) Two‐time treatment group‐protocol of two US applications, with a gap of 24 h between applications (US2X). After each experimental treatment, the incision was stapled, and the animals were allowed to awaken and recover. For groups 1 and 2, the tumors were examined at 4, 12, or 28 h post US application (for the control group, time was counted from the beginning of the US‐treated groups). For group 3, the tumors were evaluated 4 h after the second US application (i.e., 28 h after the first US application). At each time point, rats were euthanized, and the brains were carefully removed and placed in 4% paraformaldehyde for at least 24 h.

### 5.9 Histopathological evaluation of low frequency US on 9L gliosarcoma tumor mass

Six rats per time point were evaluated histologically for each group. Brains were embedded in paraffin to obtain coronal sections of 10 μm, then stained with H&E for histological observation to evaluate tumoral structural changes. Brain tissue was examined in a double‐blinded manner. The morphologic terminology was based on state‐of the art harmonized terminology.^48^ In addition, the affected area in the tumor was evaluated histologically, measured using ImageJ software,^49^ and compared to the total tumor area from each section. These ratio values were then plotted as a function of tumor volumes.

### 5.10 In vivo efficacy

Tumor growth was evaluated by measuring the tumor volume as a function of time. 8–14 rats per time point were evaluated for each group. A coronal section was performed at the center of the tumor, the maximum height and length of the tumor were measured, and volume was calculated using a half spheroid approximation (Equation (1)):

(1) $$V=0.5\times \frac{4\pi }{3}a^{2}c$$

where, *a* is half the total tumor length at the transverse plane, and c is the tumor depth in mm. This calculation was verified by measuring the volume using MRI (described in “Supplementary Materials”). All data are reported as the mean ± standard deviation (SD).

### 5.11 Statistical analysis

“The effect of low frequency US on cell viability *in vitro*”: statistical analysis of variance and Student's *t*‐test were applied. For all tests, a value of *p* < 0.05 was interpreted as significant; “Quantification of pain using the RGS”: statistical analysis was performed using XLSTAT software v. 2019 (www.xlstat.com). Group differences were performed by one‐way ANOVA followed by Dunnett's case comparison post‐hoc test. For all tests, a value of *p* < 0.05 was interpreted as significant; “*In vivo* efficacy”: statistical analysis of variance and Student's *t*‐test were applied in order to determine if there was a change in the tumor volume over time. For all tests, a value of *p* < 0.05 was interpreted as significant.

## AUTHOR CONTRIBUTIONS

**Joseph Kost:** Conceptualization; funding acquisition; resources; supervision; visualization; writing – review and editing. **Nitsa Buaron:** Conceptualization; data curation; formal analysis; investigation; methodology; project administration; validation; visualization; writing – original draft. **Antonella Mangraviti:** Data curation; investigation; project administration; writing – review and editing. **Yuan Wang:** Investigation. **Ann Liu:** Investigation; writing – review and editing. **Mariangela Pedone:** Investigation. **Eric Sankey:** Investigation. **Itay Adar:** Validation. **Abraham Nyska:** Formal analysis; validation. **Riki Goldbart:** Conceptualization; methodology; project administration; visualization; writing – review and editing. **Tamar Traitel:** Conceptualization; methodology; project administration; visualization; writing – review and editing. **Henry Brem:** Conceptualization; funding acquisition; resources; supervision; writing – review and editing. **Betty Tyler:** Conceptualization; funding acquisition; project administration; resources; supervision; visualization; writing – review and editing.

## FUNDING INFORMATION

This work was supported by the Binational Science Foundation (BSF, United States—Israel, 2009178) and Focal Technological Area (FTA) Program of the Israel National Nanotechnology Initiative (INNI).

## CONFLICT OF INTEREST STATEMENT

Dr. Henry Brem has research funding from NIH, Johns Hopkins University, Arbor Pharmaceuticals, Bristol‐Myers Squibb, and Acuity Bio Corp* and philanthropy. He is also a consultant for AsclepiX Therapeutics, StemGen, InSightec, Accelerating Combination Therapies*, Camden Partners*, LikeMinds, Inc*, Galen Robotics, Inc.* and Nurami Medical*. Betty Tyler has research funding from NIH and is a co‐owner for Accelerating Combination Therapies* (*includes equity or options). Dr. Henry Brem is a paid consultant to Insightec and chairman of the company's Medical Advisory Board. Insightec is developing focused ultrasound treatments for brain tumors. This arrangement has been reviewed and approved by the Johns Hopkins University in accordance with its conflict‐of‐interest policies. Dr. Joseph Kost has research funding from the Israel Ministry of Science and Technology, and Robert Bergida Fund.

### PEER REVIEW

The peer review history for this article is available at https://www.webofscience.com/api/gateway/wos/peer-review/10.1002/btm2.10660.

## Supporting information


**Data S1.** Supporting Information.

## Data Availability

The data that support the findings of this study are available from the corresponding author upon reasonable request.
