# Supplementary material for: Ultrasound inhibits tumor growth and selectively eliminates malignant brain tumor in vivo
Source: Bioeng Transl Med. 2024 Apr 1;9(5):e10660. doi: 10.1002/btm2.10660 (PMC11561836; doi:10.1002/btm2.10660)
Supplement: Supplementary file 1 — Data S1. Supporting Information. [file BTM2-9-e10660-s001.docx]

**SUPPLEMENTARY MATERIAL**

**Ultrasound inhibits tumor growth and selectively eliminates malignant brain tumor *in vivo***

Nitsa Buaron, Antonella Mangraviti, Yuan Wang, Ann Liu, Mariangela Pedone, Eric Sankey, Itay Adar, Abraham Nyska, Riki Goldbart, Tamar Traitel, Henry Brem & Betty Tyler, and Joseph Kost

**Table of Contents**

- Fig. S1 P. 1
- Supplementary Materials P. 2-4

Additional supportive images describing the structural changes observed in the tumor volumes that were smaller than 16 mm^3^. For the US1X group, images of the tumor tissue 12 hours after LFUS treatment are presented, showing edema, hemorrhage, and mineralization. For the US2X group, images showing mineralization are presented.


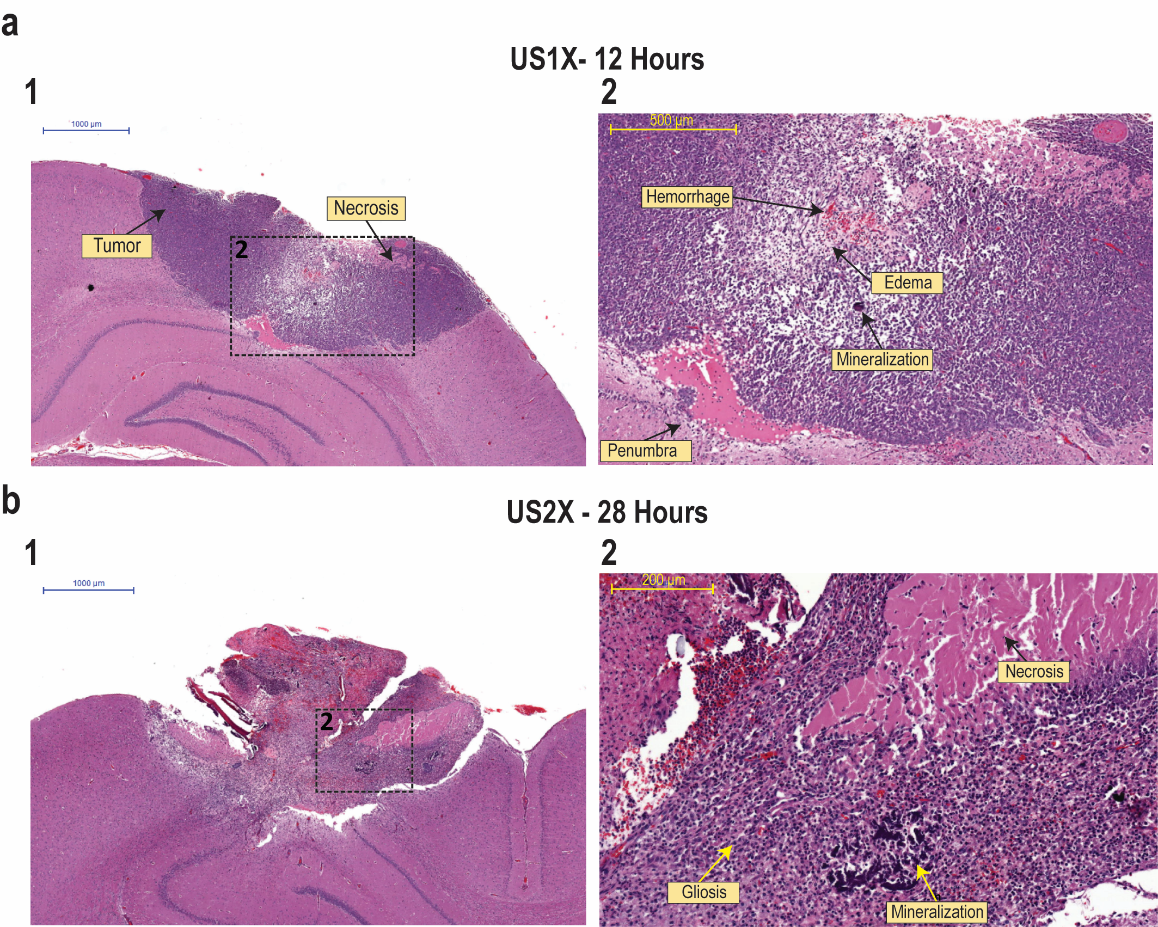


**Figure S1:** **Additional characteristics of the histopathological evaluation by different LFUS protocols.** (a) Evaluation of the structural changes from tumor treatment with the US1X protocol: different magnifications of coronal brain section of a rat bearing a 15 mm^3^ 9L tumor 12 hours post-insonation. Histological characteristics are marked within the images. Scale bar=1000µm (left image) and 500µm (right image). (b) Evaluation of the structural changes resulting from treating the tumors with the US2X protocol: different magnifications of coronal brain section of rat bearing a 9 mm^3^ 9L tumor after treatment with US2X. Histological characteristics are marked within the images. Scale bar=1000µm (left image) and 200µm (right image).

**Supplementary Methods**

Materials and methods

Cell culture

C6 rat glioma cells, obtained from ATCC (CCL-107^™^, sex of cell: male), were grown in cell culture media DMEM (Dulbecco's Modified Eagle Medium) containing 10% (v/v) fetal bovine serum, 1% (v/v) pen-strep, and 1% (v/v) L-glutamine in uncoated flasks at 37^o^C under 5% CO_2_ atmosphere. Cells were passaged every 72-96 hours. Materials were purchased from Biological Industries (Kibbutz Beit-Haemek, Israel).

The effect of low frequency US on cell viability *in vitro*

To test for US effect on cell growth, cells were seeded at a density of 100,000 cells/mL and grown to ~70% confluence in a 24-well plate. The plate was placed on top of a water-filled reservoir, 3 cm above the ultrasonic 13 cm microplate horn (20 kHz Misonix – ultrasonic liquid processor S4000-010, max intensity 600W) (Figure 1a). US was applied at intensities between 0.38 W/cm^2^ to 0.6 W/cm^2^ in a continuous mode for durations of 10 or 20 seconds. After US application, cells were incubated for 1 hour and then an MTT (Thiazolyl Blue Tetrazolium Blue) viability assay was performed. Briefly, media was replaced with 250 µL starvation medium (5% (V/V) serum, 1% (V/V) L-glutamine, and 1% (V/V) pen-strep in DMEM). 25 μL of MTT reagent (Sigma-Aldrich Inc.) was added and the plate was mixed gently. After 2 hours of incubation, 500 μL solution of isopropanol (Sigma-Aldrich Inc.) containing 0.04N HCl (Sigma-Aldrich Inc.) was added, and the plate was wrapped in aluminum foil and placed in the sterile hood overnight. Absorbance was measured 24 hours later at 570 nm wavelength using an ELISA plate reader (Bio TEK instruments, ELX 808). Results were expressed as viability percentage relative to untreated cells.

Animals

Female Fischer 344 rats, weighing 125–175 g each (Harlan Bioproducts, Indiana, IN) were housed in standard facilities and provided with ad libitum access to food and water. Female Fischer 344 rats were chosen for this set of studies since the 9L tumor is syngeneic in this strain. We have not found a difference in tumor growth rate between males and females using this tumor line. The 9L tumor model has been used as the basis for the preclinical work for both Gliadel, FDA-approved chemotherapeutic eluting wafers implanted intracranially for the treatment of malignant glioma, and OncoGel, a chemotherapeutic eluting thermosensitive paste^44–46^. While no tumor model fully recapitulates human malignant glioma, the 9L model has an aggressive growth pattern with short median survival and is notoriously difficult to treat. The policies and guidelines of the Johns Hopkins University Animal Care and Use Committee were followed throughout the study under an Animal Care and Use Committee-approved protocols (animal protocol numbers: RA19M38 and RA20M97), all animal studies meet ARRIVE guidelines and include randomization as well as sample size justification and statistical power analyses.

Intracranial burr hole placement

Rats were anesthetized with a 0.4 mL/body weight intraperitoneal injection of a stock solution containing ketamine HCl (75-100 mg/Kg, Sigma-Aldrich Inc.), xylazine (5-10 mg/Kg, Sigma-Aldrich Inc.). The head was shaved and prepared with alcohol and prepodyne solution (DeLaval Inc.), and a midline scalp incision was made, exposing the sagittal and coronal sutures. A 3 mm burr hole was made in the skull, 5 mm posterior and 3 mm to the right of the bregma, using an electric drill.

Low frequency US *in vivo* experimental setup

For US evaluation, rats were placed in a custom-made stand where their head was stabilized, and the stand could be lifted up and down in order to fix the height of the US probe. A cylinder (diameter of 1.5 cm) was placed on top of the head and the US probe was inserted into the cylinder over the burr hole. The probe height was fixed at 2 mm above the drilled hole in the skull (Figure 2b). Next, the cylinder was filled with US gel (3.5 mL) and US was applied. During US application, the gel was constantly replaced (in a continuance form, the probe was always immersed in the gel) in order to keep the temperature and the gel properties constant. 20 kHz US (Q125 Sonicator, Qsonica L.L.C, Newtown, CT, USA) with a probe tip diameter of 3.2 mm was used. The US was applied in continuous mode.

Quantification of pain using the Rat Grimace Scale (RGS)

Rats were monitored for quantification of pain using the RGS according to a published method ^47^. Accordingly, rats were monitored daily for 7 days, for pain-related facial “action units” as defined by the RGS. The four action units included orbital tightening, nose/cheek flattening, ear changes, and whisker change. Scoring was performed according to the description and guiding figures described in the published method ^47^. The scoring range was 0-2, where a score of “0” indicated that the action unit was absent, a score of “1” indicated a moderate appearance of the action unit, and a score of “2” indicated an obvious appearance of the action unit. Monitoring was performed by digital video; rats were placed separately in a cubical transparent box on a table and were filmed via digital video camera (Canon HD Camcorder VIXIA HV40) for 20-30 minutes while making sure clear head frames were acquired. Next, facial images were acquired manually from the digital videos. We used Pinnacle Studio^TM^ 14 video editing software to create short videos of the clear head frames of each rat and then we used IrfanView software to extract images from these videos. For RGS scoring, a single most suitable image was manually selected.

RGS scoring was performed as follows: images were deidentified and organized randomly, and then given to six coders (postdoctoral, graduate or undergraduate students) for blinded image scoring. Pain was quantified by averaging the action unit scores across coders. The variability within each group was described by standard error of the mean (SEM).

Surgery – 9L gliosarcoma tumor inoculation

Rats were intracranially implanted with 9L gliosarcoma, which was maintained and passaged every 2-3 weeks in the flanks of carrier rats. For surgical intracranial implantation, the tumor was removed from the carrier animal, cut into 2 mm^3^ pieces, and placed in sterile 0.9% saline on ice. A burr hole was made in the skull, as described above, and under microscopic magnification a dural opening was made through the burr hole. A small area of cortex and white matter was resected. Once hemostasis was achieved, a single tumor piece (2 mm^3^) was placed into the resection cavity. The skin was then closed with surgical staples. All surgical procedures were performed using standard sterile techniques.

*In vivo* efficacy

Tumor growth with and without US application was evaluated by measuring the tumor volume as a function of time. 8-14 rats per time point were evaluated for each group. For volume measurements, a coronal section was performed at the center of the tumor, the maximum height and length of the tumor were measured, and volume was calculated using a half spheroid approximation (equation 1):

(1) *V =*$0.5\cdot\frac{4\pi}{3}a^{2}c$

where, a is half the total tumor length at the transverse plane, and c is the tumor depth in mm. This calculation was verified by measuring the volume of several brains using MRI, scanning the brains before sectioning, and summing all T2-weighted images of hyperintensity lesion areas. The MRI volume calculations were found to correlate with the volume calculated using the half spheroid approximation with less than 10% bias.
